# Wind tunnel experimental calibration of hemispherical 7-hole probe pressure–velocity parametric equation

**DOI:** 10.1038/s41598-022-16929-2

**Published:** 2022-07-27

**Authors:** Tao Yao, Shu-dao Zhou, Song Ye, Yang-chun Zhang

**Affiliations:** 1 College of Meteorology and Oceanography, National University of Defense Technology, Changsha, 410073 China; 2 Collaborative Innovation Center on Forecast and Evaluation of Meteorological Disasters, Nanjing University of Information Science and Technology, Nanjing, 210044 China

**Keywords:** Fluid dynamics, Techniques and instrumentation

## Abstract

The multi-hole probe can measure the velocity and three-dimensional direction of the flow field at the same time, so it is often used to measure the three-dimensional flow field. Compared with other flow field measuring instruments, the multi-hole probe has stronger environmental adaptability and stability, and can better measure the three-dimensional flow field of the middle atmosphere. Therefore, a hemispherical 7-hole probe was designed, a pressure–velocity parameterized equation was established based on the theory of flow around a sphere, and a new calibration method was developed based on this. The calibration is carried out in a subsonic low speed wind tunnel, multiple combinations of flow parameters (inflow velocity and flow angles) are adjusted during the calibration. The results are compared with the numerical simulation results, both are quite close, with a speed measurement deviation of less than 5% and an angle measurement deviation of less than 1°. Our results establish the practicality of the hemispherical 7-hole probe and the simplified calibration procedure, both of which improve calibration efficiency and lower probe calibration costs.

## Introduction

The middle atmosphere generally refers to the atmosphere above the earth's surface at a height of 10–100 km, including the upper troposphere, stratosphere, middle layer, and lower thermosphere^1^. It is particularly essential in the study of global weather systems because it is an important part of the earth's atmosphere. The status of the flow field in the middle atmosphere has a direct impact on the aircraft flight, but it also has a significant impact on the movement of higher and lower atmospheres^2,3^ although in-situ measurement methods are still lacking^4,5^. Hot-wire anemometers, particle image velocimetry, laser Doppler velocimetry, and other instruments capable of monitoring three-dimensional flow fields are not suited for measuring three-dimensional flow fields in the intermediate atmosphere. Even though hotwire constant temperature anemometry (CTA) has a high temporal resolution, hotwire probes have low mechanical robustness. And the hot-wire anemometer sensor is too fragile and not suitable for high dynamic measurement. While the particle image anemometer’s imaging particles will cause interference, and the laser Doppler anemometer is susceptible to interference from complicated weather. The multi-hole probe is straightforward to design and is extremely adaptive to its surroundings. When utilized under harsh conditions multi-hole pressure probes, on the other hand, are inexpensive to produce and simple to use. To compensate for the lack of in-situ measurement data, it can be mounted on a meteorological rocket for in-situ measurement of the three-dimensional flow field of the middle atmosphere. In addition, multi-hole probe can also be used in turbines, wakes and other scenarios that need to measure three-dimensional flow fields.

It is important to remember that relevant and reliable measurement data may only be produced if the probe is calibrated under typical conditions before use. Because of the multi-hole probe's processing flaws, each probe must be calibrated before use. At present, the calibration of probes are most non-nulling, which means they convert pressure into a dimensionless pressure coefficient based on the pressure difference of the opposite hole, create a calibration data set, and compare it to the calibration data set to determine the velocity and direction of the unknown flow field^6^. As a result, the density of the data set and the inversion calculation method have a direct relationship with the flow field measurement accuracy which is a time-consuming operation. The different concept of pressure coefficient definitions^7,8^ and zoning^9^ can broaden the multi-hole probe's calibration range. The inversion calculation methods include direct interpolation^9–11^, least squares polynomial curve fitting^12,13^, and the recently popular neural network method^14,15^. Guangxing Wu has also proposed a case where a hole is blocked which improves the robustness of the multi-hole probe^16^. Although these methods can accurately measure the flow field, they use the probe as a black box. A large number of wind tunnel experiments are required to establish the calibration data set, which makes the calibration process cumbersome. Therefore, based on the principle of the flow around the sphere, a pressure–velocity parameterized equation that directly relates the hole pressure to the flow field velocity is established, and the probe is calibrated in a low-speed wind tunnel, which verifies the feasibility of this theoretical calibration method. It provides a new idea for simplifying the probe calibration process.

## Probe design and wind tunnel experimental device

### 7-hole probe structure design

**Figure 1:**
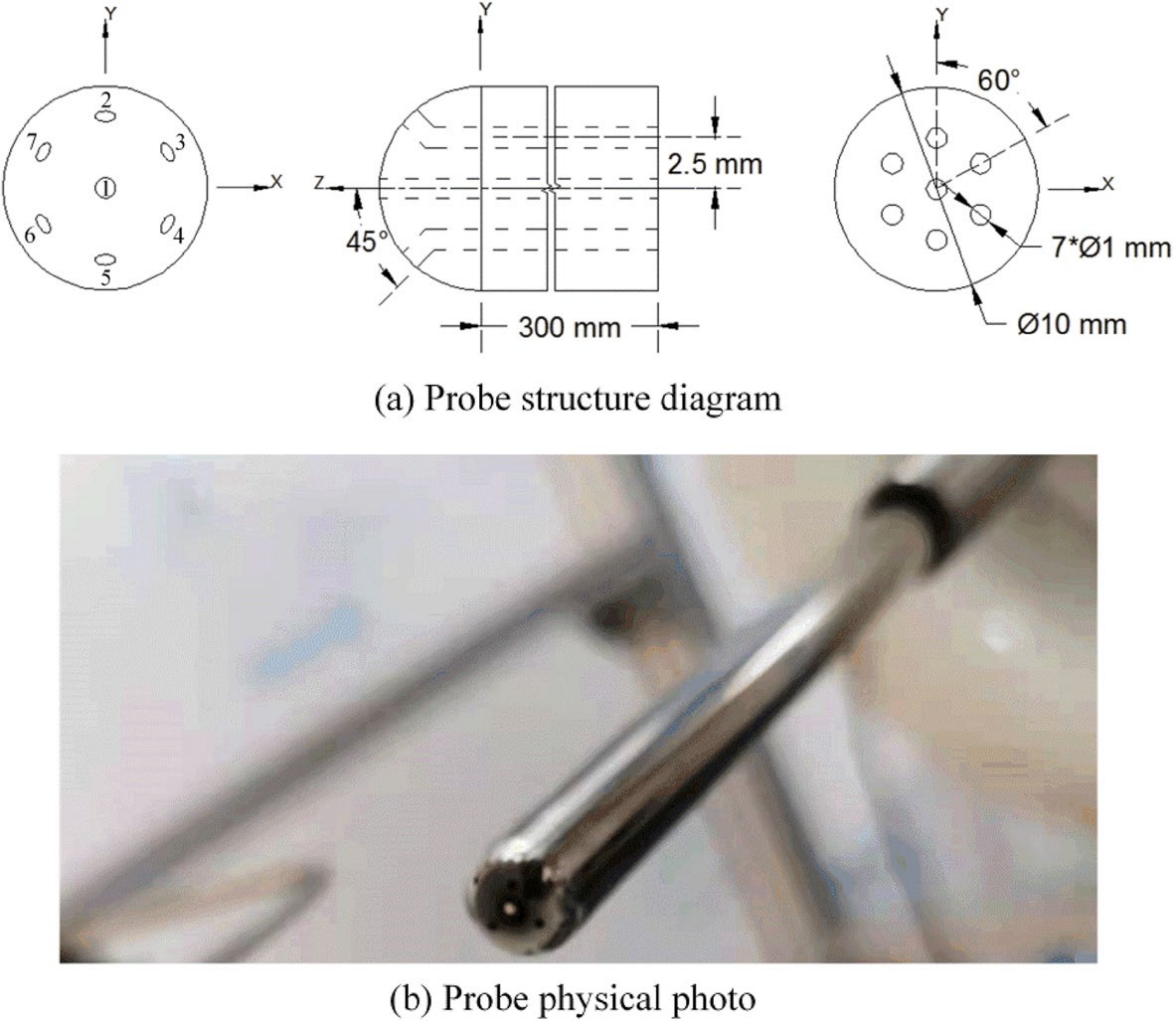
(**a**) Schematic diagram of the 7-hole probe structure, (**b**) physical diagram of the 7-hole probe.

Theoretically, the probe head can have any shape, but spherical and hemispherical probes are not sensitive to Reynolds number^17^, so they are more suitable for large speed ranges. Furthermore, the hemispherical head makes it simple to create a parametric model of pressure velocity direct correlation based on the flow around the sphere, so we designed a hemispherical 7-hole probe. The probe structure is shown in Fig. 1a. The head and rod have a diameter of 10 mm, with the central hole being the No. 1 hole and the other 6 holes evenly spaced around it. The holes are perpendicular to the probe surface to reduce airflow interference with the flow field in the hole^18^, the probe shaft is 300 mm long, and the opening direction is 45° to the center of the sphere. The physical photograph of the probe is shown in Fig. 1b.

**Figure 2:**
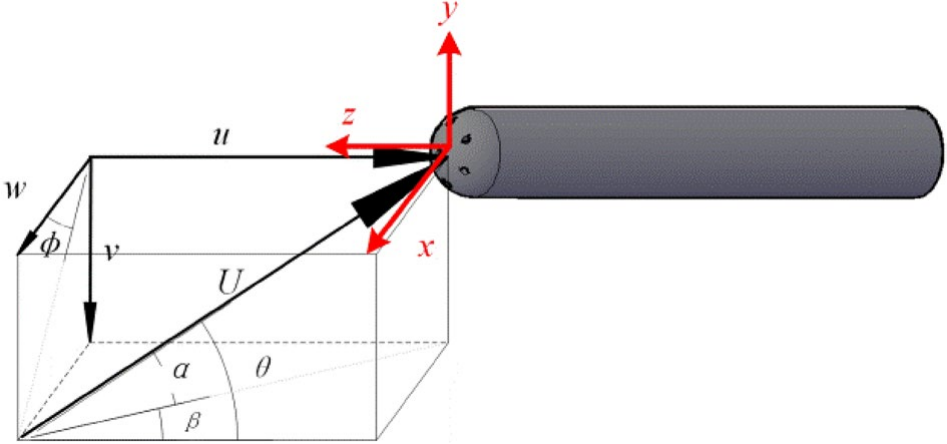
Cartesian coordinate system for the 7-hole probe.

A cartesian coordinate system for the 7-hole probe, as shown in Fig. 2, is created to express the orientation of the incoming flow and the probe. The z-axis is the axis that runs from the probe's tail to its tip, the y-axis is the direction that runs from hole 1 to hole 2, and the coordinate system's center is the hemispherical head's center. In Fig. 2, *U* is the incoming flow velocity, *u*, *v* and *w* are the velocity components in three directions. *α* is the attack angle and *β* is the sideslip angle, which represents the angle between the probe and the incoming flow on the projection plane. *θ* is the angle of pitch and *ϕ* is the roll angle, which represents the azimuth of the flow on the *x–y* plane relative to the probe. The relationship between the angles is given by:

1 $$ \left\{ \begin{gathered} u = U\cos \alpha \cos \beta = U\cos \theta \hfill \\ v = U\sin \alpha = U\sin \theta \sin \phi \hfill \\ w = U\cos \alpha \sin \beta = U\sin \theta \cos \phi \hfill \\ \end{gathered} \right. $$

### Experimental design

**Figure 3:**
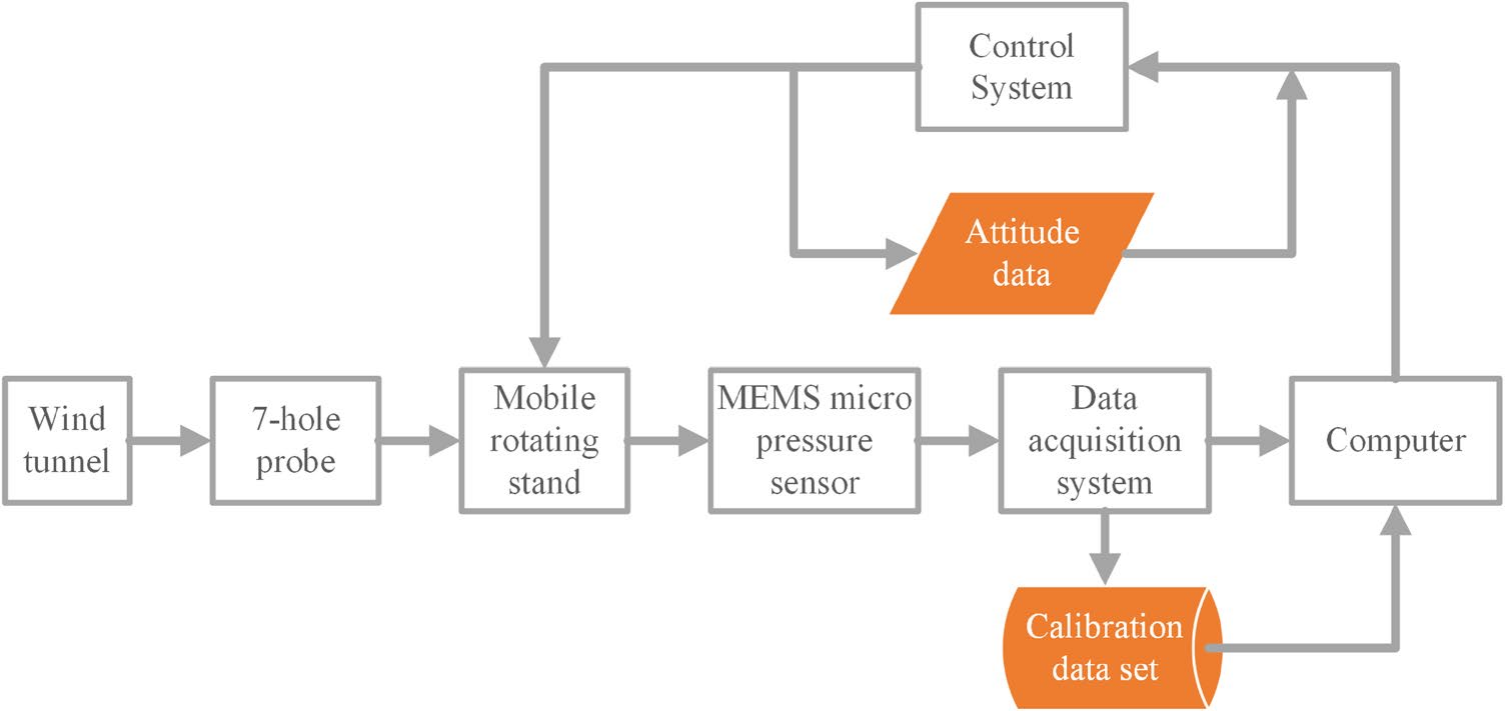
Block diagram of the test system.

**Figure 4:**
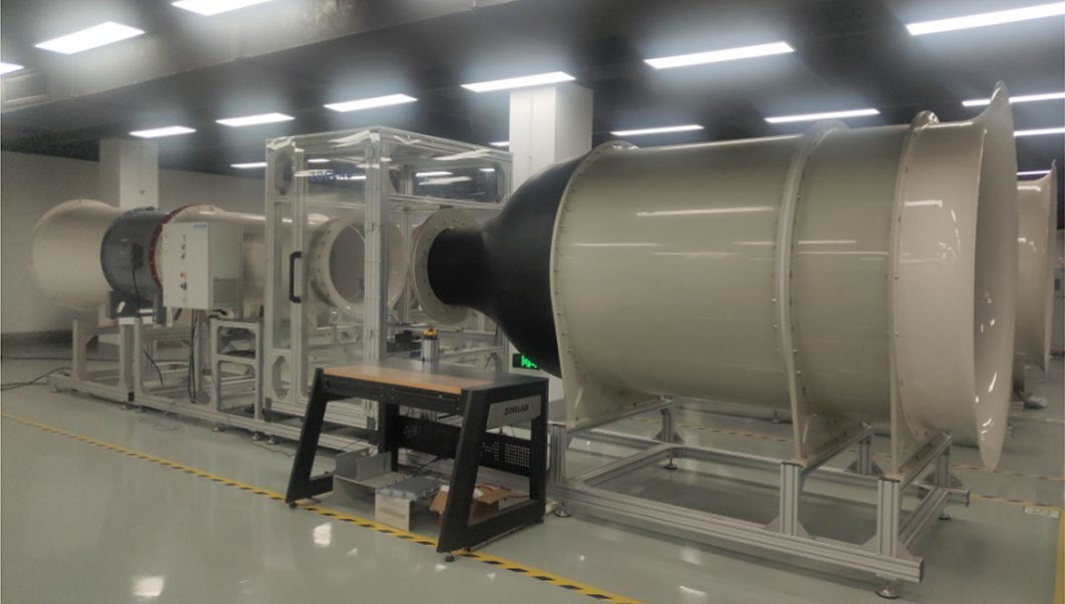
40 m/s straight open wind tunnel.

Figure 3 shows the design of the multi-hole probe calibration and test system's block diagram. The calibration and test experiments are carried out in a 40 m/s straight open wind tunnel. The wind tunnel is shown in Fig. 4. The test section of wind tunnel has a diameter of 0.6 m, a length of 1 m, a wind speed range of 0.2–40 m/s. Before calibration, the uniformity of the flow field is tested, the turbulence is less than 0.5%, the center point is less than 0.1%, and the air flow deflection angle is less than 1°. The static pressure and total pressure of 7 holes and Pitot tube are measured using a MEMS-based micro-pressure sensor with a measurement range of 0–4 kPa and measurement precision of 0.1%. In addition, the air pressure, temperature, and humidity in the experimental chamber and the ambient are monitored in real-time.

**Figure 5:**
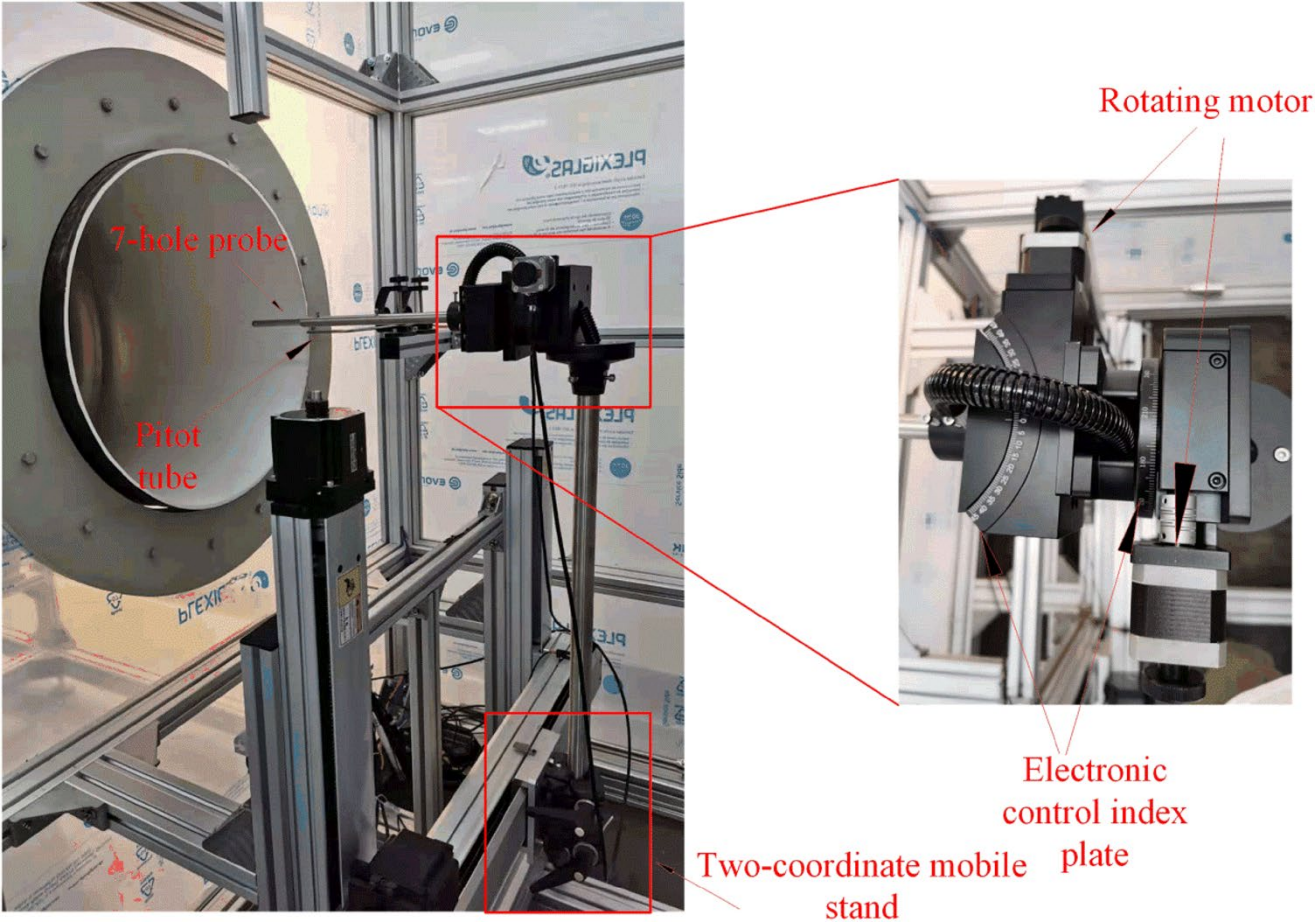
Two-coordinate mobile calibration system.

Figure 5 shows the structure of the two-coordinate mobile support. The 7-hole probe is fixed on the support through the fixing sleeve. With a step length of 1 mm, the device may translate horizontally and vertically. The motor allows the probe to revolve 360° about its axis and 45° in the horizontal direction, with the root of the probe as the center. The accuracy of the motor rotation angle is 0.1°. The Pitot tube is installed on the side of the wind tunnel, 0.1 m away from the wind tunnel wall. During the measurement, the angle of pitch *θ* between the incoming flow and the probe is realized by controlling the left and right angle changes, the azimuth angle *ϕ* is controlled by the rotating motor, and the Pitot tube measures the total pressure and static pressure of the incoming flow.

## Calibration method

### Traditional calibration method

Currently, the measurement of flow field velocity and direction by the 7-hole probe is primarily based on the report by Gerner, Maurer, and Gallington et al.^12,13^, who used the pressure difference between opposite holes to construct a dimensionless pressure coefficient only related to the direction and independent of velocity. A total of four pressure coefficients need to be defined: pitch coefficient, yaw coefficient, total pressure coefficient, and dynamic pressure coefficient. Use the wind tunnel calibration data set to link the pressure coefficient to the fluid velocity and direction. The pressure of central hole 1 is up to the total pressure, which can be regarded as the local total pressure, and the average pressure of 2–7 holes can be regarded as the local static pressure, according to the number of 7-hole probe holes illustrated in Fig. 1. According to the pressure difference of relative hole, three groups of pressure coefficients related to the direction can be defined:

2 $$ \left\{ \begin{gathered} C_{\alpha 1} = \frac{{P_{5} - P_{2} }}{{P_{1} - \overline{P}_{2{-}7} }} \hfill \\ C_{\alpha 2} = \frac{{P_{4} - P_{7} }}{{P_{1} - \overline{P}_{2{-}7} }} \hfill \\ C_{\alpha 3} = \frac{{P_{3} - P_{6} }}{{P_{1} - \overline{P}_{2{-}7} }} \hfill \\ \end{gathered} \right. $$

where, Pi (i = 1,2,…, 7) is the pressure value measured at the *i*-th hole; $\overline{P}_{2{-}7}$ is the average pressure value of No. 2–7 hole. According to the position distribution of the hole and its contribution to the pitch angle and yaw angle, the pitch coefficient $C_{\alpha }$ and yaw coefficient $C_{\beta }$ can be defined. In addition, the total pressure coefficient *C*_*o*_ and dynamic pressure coefficient *C*_*q*_ need to be defined as follows^12^:

3 $$ \left\{ \begin{gathered} C_{\alpha } = \frac{1}{3}(2C_{\alpha 1} + C_{\alpha 2} - C_{\alpha 3} ) \hfill \\ C_{\beta } = \frac{1}{\sqrt 3 }(C_{\alpha 2} + C_{\alpha 3} ) \hfill \\ C_{O} { = }\frac{{P_{1} - P_{O} }}{{P_{1} - \overline{P}_{2{-} 7} }} \hfill \\ C_{q} { = }\frac{{P_{1} - \overline{P}_{2{-}7} }}{{P_{O} - P_{\infty } }} \hfill \\ \end{gathered} \right. $$

where $P_{O}$ and $P_{\infty }$ are total pressure and static pressure, respectively. Through polynomial fitting, the wind tunnel experimental data and pressure coefficients are connected with the velocity and direction of the flow field, and the polynomial coefficient set is established to complete the probe calibration. According to the position of the highest pressure hole, a total of seven pressure coefficient groups must be determined. It may be noted that the non-nulling calibration approach necessitates 560 measurement points, and even if the data reduction method, such as the Latin Square method^13^ is used, 252 measurement points are needed. As such the amount of data required is enormous, resulting in a very expensive probe calibration cost.

### Establishment of the pressure–velocity parameterized theoretical model

**Figure 6:**
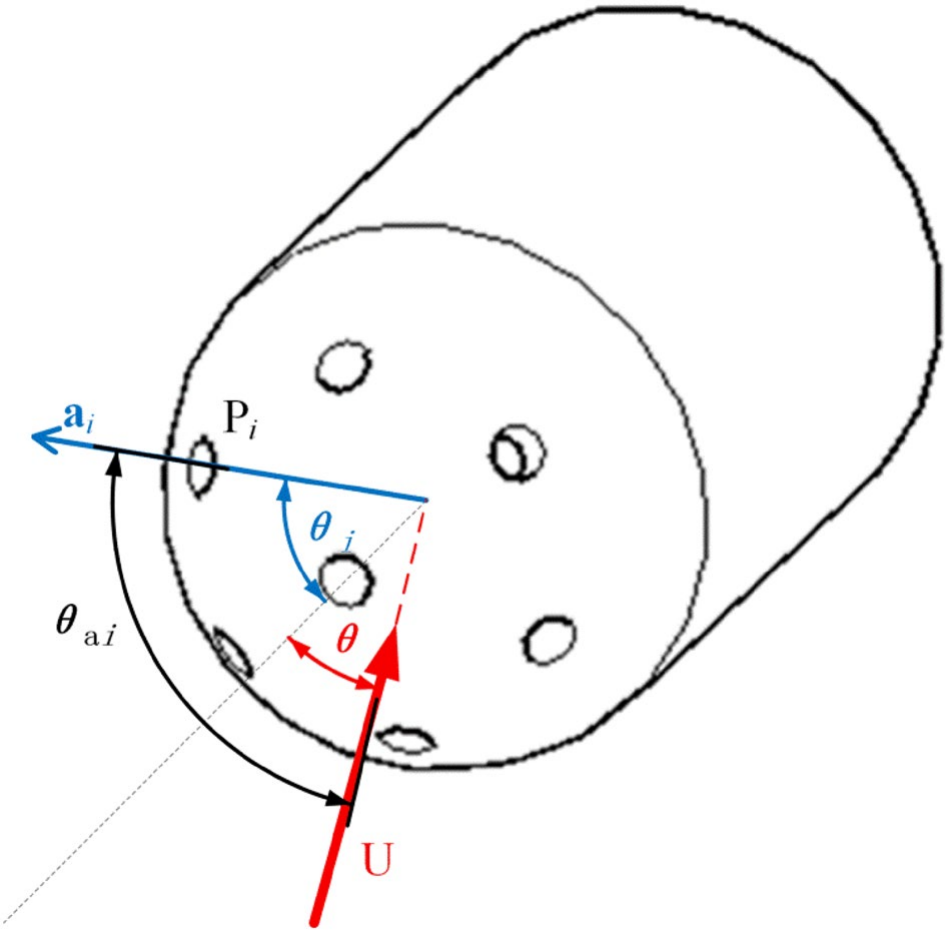
7-hole probe calibration coordinate system.

The probe is treated as a black box in the classic calibration procedure, and its calibration is entirely dependent on the wind tunnel experiment. However, this calibration method necessitates a significant number of wind tunnel tests, extending the calibration time and increasing the calibration cost. When the hemispherical 7-hole probe was measured, the hole was located in the attached flow when the incoming flow was flowing to the probe due to its hole angle of 45°. Therefore, the relationship between the pressure in the hole and fluid velocity can be established according to Bernoulli's principle. The rectangular coordinate system of the 7-hole probe is established, as shown in Fig. 6. $\theta_{i}$ being the angle between the *i*-hole and the central hole, $\theta$ is the angle between the incoming flow and the central hole, and $\theta_{ai}$ is the angle between the *i*-hole and the incoming flow. For a sphere in the flow field, the velocity of a point on the sphere can be regarded as a function of the total angle *θ* from the stagnation point. The speed of any point on the surface of the sphere can be expressed as:

4 $$ V(\theta ) = \frac{3}{2}U_{\infty } \sin \theta $$

where $U_{\infty }$ is the speed of incoming flow. According to the Bernoulli equation, the pressure distribution on the sphere can be obtained as:

5 $$ p_{i} + \frac{1}{2}\rho V^{2} = p_{s} + \frac{1}{2}\rho U_{\infty }^{2} $$

where *p*_*i*_ is the pressure at point *i* on the spherical surface, and *p*_*s*_ is the static pressure.

6 $$ f_{i} (\theta ) = \frac{{p_{i} - p_{s} }}{q} $$

The dimensionless pressure coefficient at any point on the spherical surface can then be obtained by simultaneous solution of expressions (4)–(6) as:

7 $$ f_{i} (\theta ) = \frac{9}{4}\cos^{2} \theta - \frac{5}{4} $$

In Fig. 6, ${\mathbf{a}}_{i}$ is the vector represented by *i*-hole in the cartesian coordinate system, so its direction vector can be expressed as:

8 $$ {\mathbf{a}} = r\sin \theta_{i} {\mathbf{i}} + r\sin \theta_{i} \sin \phi_{i} {\mathbf{j}} + r\sin \theta_{i} \cos \phi_{i} {\mathbf{k}} $$

The direction vector of the incoming flow can be expressed as:

9 $$ {\mathbf{U}} = U\cos \theta {\mathbf{i}} + U\sin \theta \sin \phi {\mathbf{j}} + U\sin \theta \cos \phi {\mathbf{k}} $$

Then through these two vectors, the angle between the incoming flow and the *i*-hole can be expressed as:

10 $$ \cos \theta_{ai} = \frac{{{\mathbf{U}} \cdot {\mathbf{a}}}}{{\left| {\mathbf{U}} \right|\left| {\mathbf{a}} \right|}} = \cos \theta \cos \theta_{i} + \sin \theta \sin \phi \sin \theta_{i} \sin \phi_{i} + \sin \theta \cos \phi \sin \theta_{i} \cos \phi_{i} $$

Substituting Eqs. (10), (6), and (1) into Eq. (7), the relationship between pressure and velocity at 7 holes for any incoming flow is given as :

11 $$ \frac{{p_{i} - p_{s} }}{\rho } = A_{i} u^{2} + B_{i} v^{2} + C_{i} w^{2} + D_{i} uv + E_{i} uw + F_{i} vw $$

where

12 $$ \left\{ \begin{gathered} A_{i} = \frac{9}{8}\cos^{2} \theta_{i} - \frac{5}{8} \hfill \\ B_{i} = \frac{9}{8}\sin^{2} \theta_{i} \sin^{2} \phi_{i} - \frac{5}{8} \hfill \\ C_{i} = \frac{9}{8}\sin^{2} \theta_{i} \cos^{2} \phi_{i} - \frac{5}{8} \hfill \\ D_{i} = \frac{9}{4}\sin \theta_{i} \cos \theta_{i} \sin \phi_{i} \hfill \\ E_{i} = \frac{9}{4}\sin \theta_{i} \cos \theta_{i} \cos \phi_{i} \hfill \\ F_{i} = \frac{9}{4}\sin^{2} \theta_{i} \cos \theta_{i} \sin \phi_{i} \hfill \\ \end{gathered} \right. $$

Equation (11) is a parametric equation that connects the flow field velocity with the pressure in the hole. When the hole angle *θ* is determined, the parameters $A_{i} \sim F_{i}$ are constant. Considering the machining and measurement errors, the parameters need to be re-determined during calibration. During calibration, for selected 6 test points with different azimuth and angle of attack, and substituting the measured pressure data into Eq. (11) one obtains the following equations:

13 $$ \left( \begin{gathered} \left( {\frac{{p_{i} - p_{s} }}{\rho }} \right)_{1} \\ \left( {\frac{{p_{i} - p_{s} }}{\rho }} \right)_{2} \\ \vdots \\ \left( {\frac{{p_{i} - p_{s} }}{\rho }} \right)_{6} \\ \end{gathered} \right) = \left( {\begin{array}{*{20}c} {u_{1}^{2} } &\quad  {v_{1}^{2} } &\quad  {w_{1}^{2} } &\quad  {uv_{1} } &\quad  {uw_{1} } &\quad  {vw_{1} } \\ {u_{2}^{2} } &\quad  {v_{2}^{2} } &\quad  {w_{2}^{2} } &\quad  {uv_{2} } &\quad  {uw_{2} } &\quad  {vw_{2} } \\ \vdots &\quad  \vdots &\quad  \vdots &\quad  \vdots &\quad  \vdots &\quad  \vdots \\ {u_{6}^{2} } &\quad  {v_{6}^{2} } &\quad  {w_{6}^{2} } &\quad  {uv_{6} } &\quad  {uw_{6} } &\quad  {vw_{6} } \\ \end{array} } \right) \times \left( {\begin{array}{*{20}c} {A_{i} } \\ {B_{i} } \\ {C_{i} } \\ {D_{i} } \\ {E_{i} } \\ {F_{i} } \\ \end{array} } \right) $$

The matrix form is given by:

14 $$ {\mathbf{P}}_{i} = {\mathbf{X}} \times {\mathbf{b}}_{i} $$

The parameter matrix can be obtained from the following formula:

15 $$ {\mathbf{b}}_{i} = \left( {{\mathbf{X}}^{T} {\mathbf{X}}} \right)^{ - 1} {\mathbf{X}}^{T} \times {\mathbf{P}}_{{\text{i}}} $$

where

16 $$ {\mathbf{P}}_{i} = \left( \begin{gathered} \left( {\frac{{p_{i} - p_{s} }}{\rho }} \right)_{1} \\ \left( {\frac{{p_{i} - p_{s} }}{\rho }} \right)_{2} \\ \vdots \\ \left( {\frac{{p_{i} - p_{s} }}{\rho }} \right)_{6} \\ \end{gathered} \right) $$

17 $$ {\mathbf{X}} = \left( {\begin{array}{*{20}c} {u_{1}^{2} } &\quad  {v_{1}^{2} } &\quad  {w_{1}^{2} } &\quad  {uv_{1} } &\quad  {uw_{1} } &\quad  {vw_{1} } \\ {u_{2}^{2} } &\quad  {v_{2}^{2} } &\quad  {w_{2}^{2} } &\quad  {uv_{2} } &\quad  {uw_{2} } &\quad  {vw_{2} } \\ \vdots &\quad  \vdots &\quad  \vdots &\quad  \vdots &\quad  \vdots &\quad  \vdots \\ {u_{6}^{2} } &\quad  {v_{6}^{2} } &\quad  {w_{6}^{2} } &\quad  {uv_{6} } &\quad  {uw_{6} } &\quad  {vw_{6} } \\ \end{array} } \right) $$

18 $$ {\mathbf{b}}_{i} = \left( {\begin{array}{*{20}c} {A_{i} } \\ {B_{i} } \\ {C_{i} } \\ {D_{i} } \\ {E_{i} } \\ {F_{i} } \\ \end{array} } \right) $$

### Calibration and measurement process

**Figure 7:**
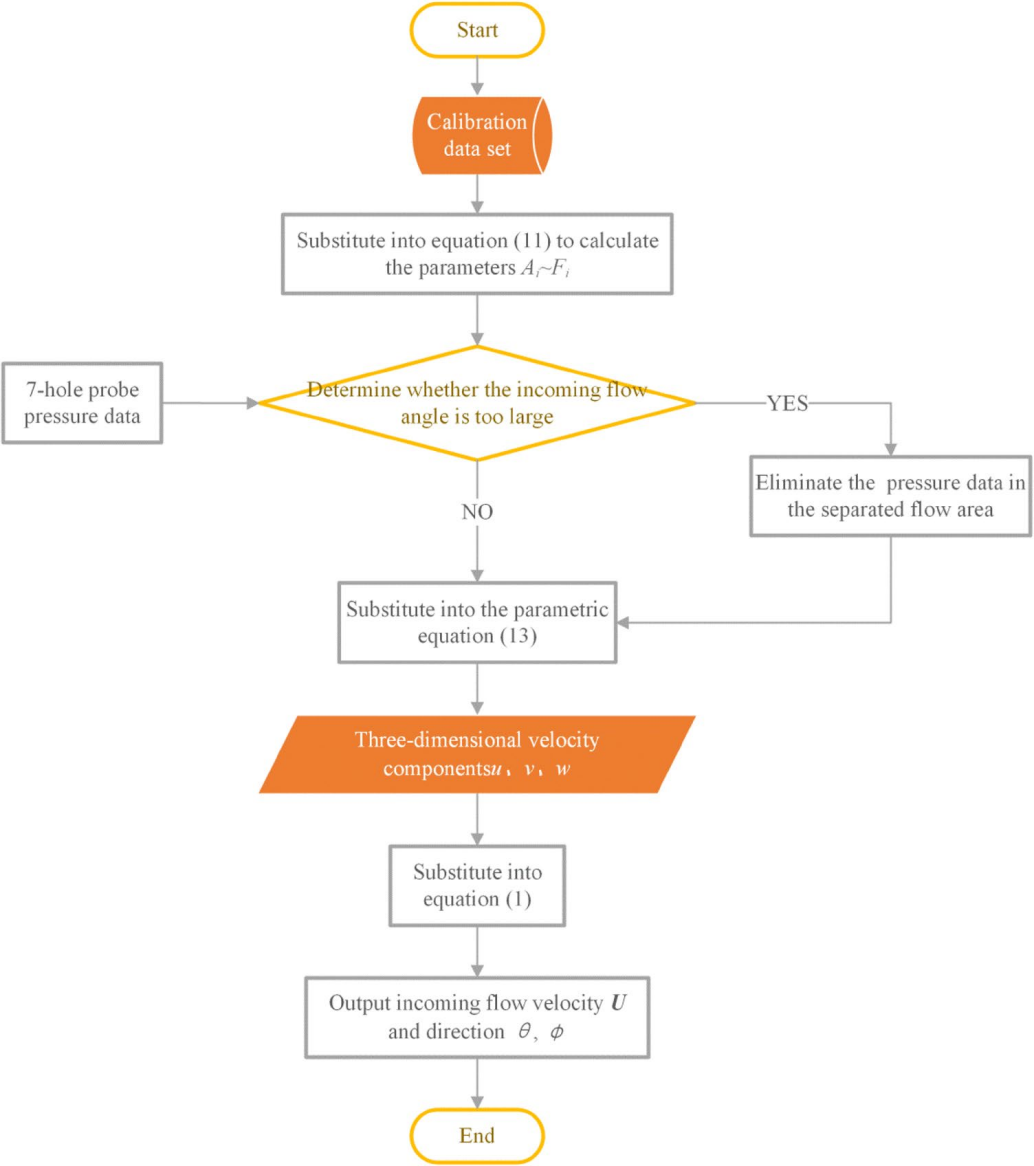
Flow chart of probe calibration measurement.

Before measuring the flow field, the probe needs to be placed in a uniform flow field for calibration The main calibration process is as follows (see Fig. 7 for the flow diagram):

1. When the flow field is stable, adjust the initial location of the probe by altering the pitch and yaw angle to take the pressure of the 6 peripheral holes equal.
2. To obtain a calibration data set, change the velocity of the flow field and the angle of the probe, and record the pressure of 7 holes under flow conditions of known velocity and direction.
3. According to the calibration pressure data set, select 6 groups of pressure data measured at different angles of attack and azimuth and substitute them into formula (11) to calculate parameter $A_{i} \sim F_{i}$;
4. Substitute the obtained parameter $A_{i} \sim F_{i}$ into formula (13) to obtain the pressure–velocity parameterized equation;
5. Check whether the pressure data from 7 holes is out of range during the measurement;
6. Substitute the pressure data of 7 holes into formula (13) to calculate the three-dimensional velocity components *u*, *v,* and *w*;
7. Get the speed and direction of the incoming flow according to formula (1).

## Results and discussion

### Calibration results

**Figure 8:**
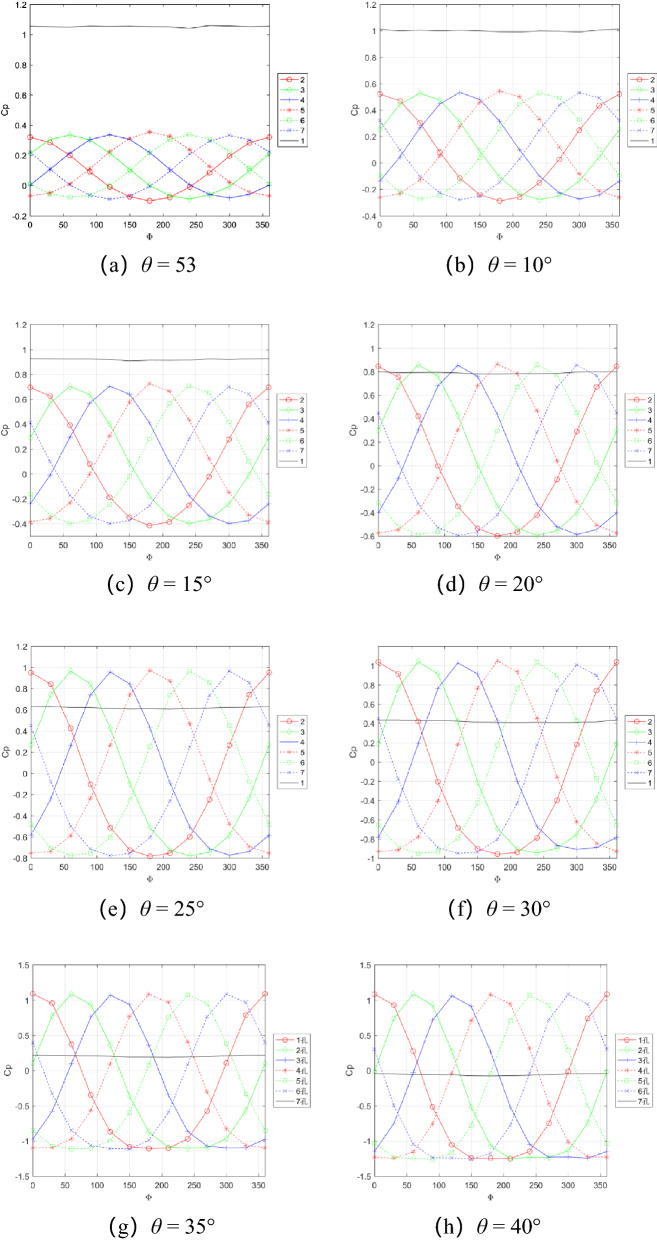
Variation curve of pressure coefficient of seven holes with roll angle at different pitch angles.

When the wind speed is 10 m/s, 20 m/s, 30 m/s, and 40 m/s, calibration trials are conducted. The variation range of pitch angle is 0°–40°, 5° interval during calibration, and the variation range of roll angle is 0°–360°, 30° interval. A qualitative analysis of the measured pressure data is carried out. Figure 8 shows the change curve of the pressure coefficient of the seven holes with the roll angle at different pitch angles. It can be seen from the figure that the 6 peripheral holes change periodically with the roll angle, and the central hole 1 has almost no change, which is consistent with the spherical distribution of the holes on the probe. However, when the pitch angle θ = 40°, the pressure coefficient curve is obviously asymmetry. When the pressure coefficient is negative, the change is very gentle, and its change is inconsistent with the change of the spherical surface pressure, indicating that the farthest hole relative to the incoming flow may be in a flow separation state at this time. With the increase of the pitch angle, the absolute value of the pressure coefficient gradually increases, while the pressure coefficient value of the central hole 1 gradually decreases to zero, which indicates that the area covered by the probe head is larger with the increase of the angle of attack. At each speed, 6 test points are selected as the calibration data set, and they are substituted into the pressure-speed parameterized equation to obtain the parameters shown in Table 1.

**Table 1:** Coefficients of pressure velocity parameterization equation.

	A	B	C	D	E	F
b_1_	0.577981	− 0.79733	− 0.80921	0.026436	− 0.02865	0.017465
b_2_	0.066534	− 0.83363	0.178752	0.026397	1.260787	− 0.00366
b_3_	0.055465	− 0.19636	− 0.50056	1.122224	0.622973	0.806135
b_4_	0.05325	− 0.23645	− 0.67323	1.100681	− 0.62316	− 0.67927
b_5_	0.080302	− 0.89324	− 0.30561	− 0.0164	− 1.29094	− 0.01824
b_6_	0.066041	− 0.26272	− 0.40607	− 1.00528	− 0.5964	0.710998
b_7_	0.089621	− 0.2882	− 0.81311	− 1.04852	0.541632	− 0.63486

**Figure 9:**
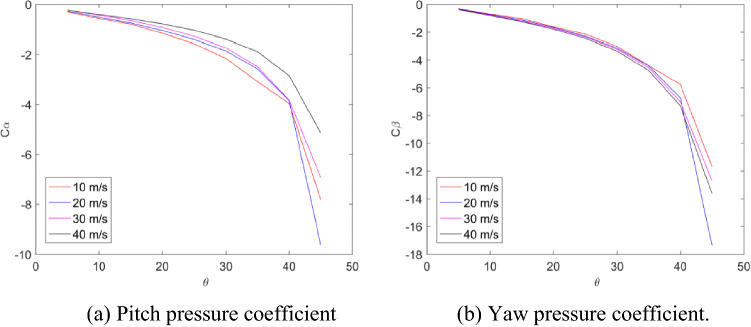
(**a**) Variation curve of pitch pressure coefficient with angle of attack. (**b**) Variation curve of yaw pressure coefficient with angle of attack.

Figure 9 shows the variation of the pitch pressure coefficient and the yaw pressure coefficient with the angle of attack at different speeds. It can be seen from the figure that when the angle of attack is less than 40°, the pressure coefficient does not change significantly with the increase of speed. When the angle of attack is greater than 40°, the pressure coefficient gap increases at different speeds, which may be due to the leeward area when the angle of attack is too large. Pores may be in separate flow regions. When the angle of attack is greater than 40°, there will be a large deviation in the measurement results. So the probe measuring range is less than ± 40°.

After calibration, an additional test point is randomly selected at different speeds to check the pressure and velocity parameter equation. The flow field inversion results are shown in Table 2.The deviation has been analyzed, and the results of the deviation are listed in Table 3. The velocity inaccuracy is around 5%, and the deviation of angle calculation is 0.5°, as can be seen from the inversion data. Compared with the deviation of the traditional methods in Table 4, it can be found that the deviation of the two methods are equivalent at 10 m/s, and the errors only increase slightly when the speed increases. However, the method based on the pressure–velocity parameterized equation requires much less calibration points than the traditional method, and the calibration process is also simpler. It demonstrates that this calibration approach can meet the requirements for 7-hole probe calibration. Although the roll angle error is large at 40 m/s, this is due to the high wind speed, and the probe shakes severely during calibration. And compared with the traditional calibration method, it reduces the test points required for the probe calibration and shortens the probe calibration process.

**Table 2:** Flow field inversion results.

	Flow field parameters				Probe inversion result			
	U_0_	u_0_	v_0_	_W0_	U	u	v	w
1	10	9.6593	2.2414	1.2941	10.12226	9.7632	2.3353	1.2986
2	20	19.3185	4.48288	2.58819	18.77712	17.8729	5.0146	2.8273
3	30	28.9778	6.72432	3.88229	29.28921	28.5301	5.6511	3.4578
4	40	38.637	8.96575	5.17638	40.03017	38.6928	9.3067	4.3205

**Table 3:** Deviation of flow field inversion results.

	∆U (%)	*θ*	∆$\theta$ (deg)	*φ*	∆$\varphi$ (deg)
1	1.2236	15.31	0.31	60.92	0.92
2	6.1144	17.85	2.85	60.59	0.59
3	2.3693	13.07	1.93	58.54	1.46
4	0.0754	14.85	0.15	65.09	5.10

**Table 4:** Global deviation of traditional methods by different authors.

	∆U (%)	∆$\theta$ (deg)	∆$\varphi$ (deg)
Gerner^14^	1.4	0.78	0.72
Wu^17^	2.85	0.93	0.72

### Comparison of wind tunnel data and simulation results

**Figure 10:**
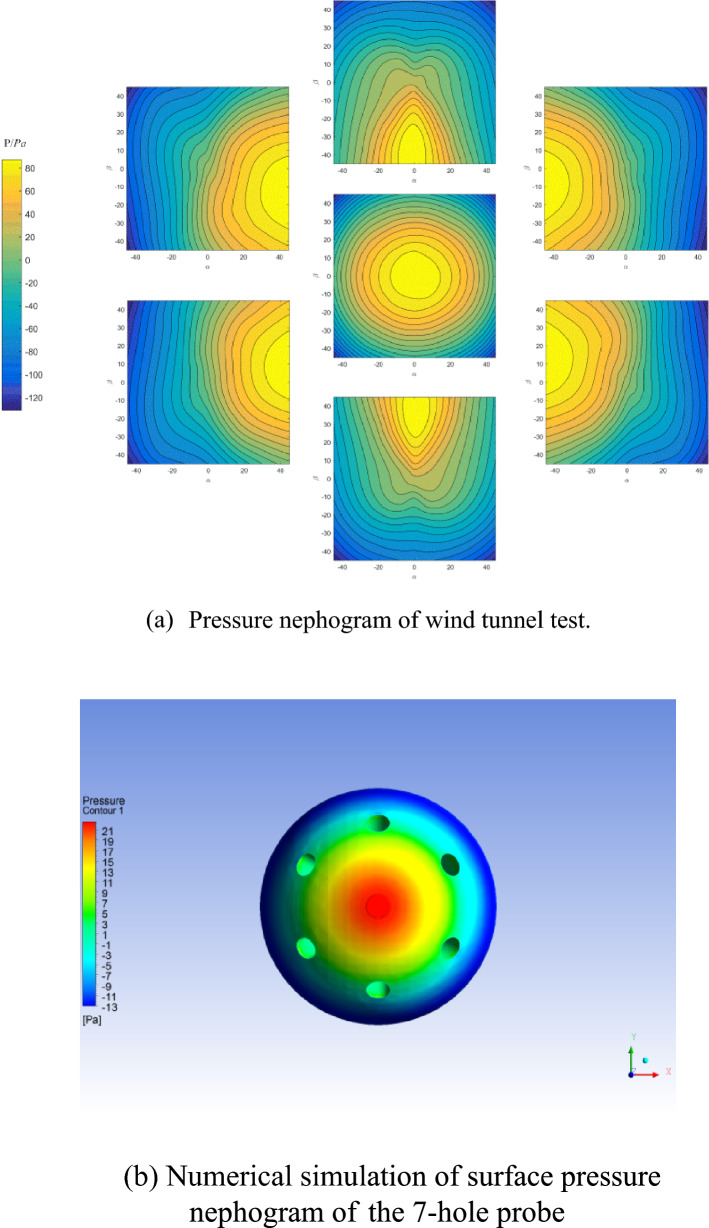
Pressure nephogram of the 7-hole probe experimental data and numerical simulation.

According to the results of the wind tunnel calibration data set, the angle of attack *θ* and azimuth angle *ϕ* change to pitch angle *α* and yaw angle *β*. The pressure data of the seven holes at 10 m/s were drawn into a pressure cloud diagram, as shown in Fig. 10a. At the same time, in the environment of 10 m/s, temperature of 273k, standard atmospheric pressure and ideal atmosphere, numerical simulation was carried out with the Spalar-Allmaras model, and the pressure distribution on the probe surface as shown in Fig. 10b was obtained. The pressure center distribution of each hole in Fig. 10a is essentially consistent with the hole position distribution, showing that the probe surface pressure distribution can be inversed after the probe calibration to obtain the incoming flow information. In Fig. 10a, the distribution of the medium value line is not as "circular" as in the numerical simulation, which is due to the measurement error produced by the experiment, which coincides with the error of the flow field inversion result.

## Conclusion

A hemispherical 7-hole probe with a diameter of 10 mm is designed and manufactured to meet the in-situ measurement of a three-dimensional flow field in the middle atmosphere. A pressure–velocity parameterized equation that directly relates the flow field velocity to the pressure in the hole is derived, and a new calibration method is proposed based on this. In the straight open wind tunnel, the hemispherical 7-hole probe is calibrated and tested; the velocity deviation of the test findings is less than 5%, and the angle deviation is less than 1°. The calibration data findings are compared to the numerical simulation cloud diagram, and the results are largely consistent, indicating that the pressure–velocity parametric equation-based calibration method can match the measurement requirements. The proposed calibration technique requires fewer calibration test points than the pressure coefficient calibration method, significantly shortening the probe calibration process time.

The 7-hole probe has only been calibrated and tested in a low-speed wind tunnel so far, with a maximum wind speed of only 40 m/s. When the velocity of the flow field increases, the number of holes in the separation flow will increase, and the measurement error may further increase. As a result, it is necessary to further study the situation at high speed.

## Data Availability

The datasets generated and analysed during the current study are not publicly available due [Project confidentiality] but are available from the corresponding author on reasonable request.
